# A Supplementary Register for Fever Nursing

**Published:** 1919-05-10

**Authors:** H. I. Bryson

**Affiliations:** Secretary, Fever Nurses' Association.


### A Supplementary Register for Fever Nursing.

The following is a copy of a letter which has been sent by the Council of the Fever Nurses' Association to the Hon. Secretary of the Medical Committee of the House of Commons:—

*State Registration of Nurses Bill.*

Sir,—It has been brought to the knowledge of the Council of the Fever Nurses' Association that during the Committee stage of this Bill it was proposed that a clause providing for the establishment of a Supplementary Register for Fever Nurses should be inserted in the Bill. The Council understands that though this proposal was not pressed in Committee, it will probably be renewed on report stage.

I am instructed by the Council to write and inform you that the Fever Nurses' Association has always, from its foundation in 1907, been against the setting up of a separate register for fever nurses in any Nurses' Registration Bill for the following reasons:—

1. Because it is quite impossible to give surgical and gynæcological training to nurses in fever hospitals to the extent that could be considered adequate for a general-trained nurse;

2. Because the institution of such a Supplementary Register would act adversely to the interests of the Nurses who would be enrolled on the General Register, by leading the public to suppose that fever-trained nurses were equal to the general-trained nurses; and

3. Because also the institution of such a Register would encourage women to enter what would be for most of them a "blind-alley" occupation.

The Council of the Fever Nurses' Association is of the opinion that the interests of the fever nurses are sufficiently safeguarded by the provisions concerning them as they stand in the Bill after it passed the Standing Committee.

I am instructed by the Council of the Fever Nurses' Association to ask you to be good enough to bring this letter before your Committee.—I am, sir, your obedient servant,

(Signed) H. I. Bryson,

May 1, 1919. Secretary, Fever Nurses' Association.

